# Associations between measures of socio-economic status, beliefs about back pain, and exposure to a mass media campaign to improve back beliefs

**DOI:** 10.1186/s12889-017-4387-4

**Published:** 2017-05-25

**Authors:** Arnela Suman, Geoffrey P. Bostick, Frederieke G. Schaafsma, Johannes R. Anema, Douglas P. Gross

**Affiliations:** 1 Department of Public and Occupational Health, Amsterdam Public Health research institute, VU University Medical Centre, PO Box 7067, 1007 MB Amsterdam, The Netherlands; 2 Department of Physical Therapy, University of Alberta, 2-50 Corbett Hall, Edmonton, T6G 2G4 Canada; 3 Research Centre for Insurance Medicine, Collaboration between AMC-UMCG-UWV-VUmc, Amsterdam, The Netherlands

**Keywords:** Social class, Socioeconomic factors, Surveys and questionnaires, Health education, Low back pain, Mass media

## Abstract

**Background:**

Low back pain (LBP) is one of the most common and costly healthcare problems worldwide. Disability from LBP is associated with maladaptive beliefs about the condition, and such beliefs can be influenced by public health interventions. While socioeconomic status (SES) has been identified as an important factor in health literacy and inequalities, not much is known about the association between SES and beliefs about LBP. Therefore, this study examined the relationship between measures of SES and the belief that one should stay active through LBP in a representative sample of the general population in Alberta, Canada. We also examined the association between measures of SES and self-reported exposure to a LBP mass media health education campaign.

**Methods:**

Population-based surveys from 2010 through 2014 were conducted among 9572 randomly selected Alberta residents aged 18–65 years. Several methods for measuring SES, including first language, education, employment status, occupation, and annual household income, were included in multivariable logistic regression modeling to test associations between measures of SES and outcomes.

**Results:**

Univariable analysis showed that age, language, education, employment, marital status, and annual household income were significantly associated with the belief that one should stay active through LBP. In multivariable analysis, income was the variable most strongly correlated with this belief (odds ratios ranged from 1.04 to 1.62 for the highest income category, *p* = 0.005). Univariable analysis for exposure to the campaign showed age, language, education, employment, and occupation to be significantly associated with self-reported exposure, while only education (*p* = 0.01) and age (*p* = 0.001) remained significant in multivariable analysis.

**Conclusions:**

Individuals with higher annual income appear more likely to believe that one should stay active during an episode of LBP. Additionally, targeted information campaigns are recalled more by low SES groups and may thus assist in reducing health disparities. More research is needed to fully understand the association between socioeconomic factors and LBP and to target campaigns accordingly.

**Electronic supplementary material:**

The online version of this article (doi:10.1186/s12889-017-4387-4) contains supplementary material, which is available to authorized users.

## Background

Socioeconomic status (SES), conceptualized as the social standing or class of an individual or group, has been identified as an important factor in health inequalities [1, 2]. People with higher SES tend to live longer, healthier lives, and suffer less from disease and disability [3, 4]. Furthermore, SES and disability appear to have a gradient relationship, with better health accompanying increases in SES [5]. Adler and Newman have proposed several pathways through which SES may influence health [6]. These include SES and environmental exposures (i.e. exposure to damaging agents in the work or home environment), SES and social environment (i.e. isolation and engagement in social networks), SES and health care (i.e. access, use, and quality of health care), and SES and behaviour and lifestyle [6].

With many pathways through which SES can influence health, many approaches to measure SES have been taken in health research. For example, level of education, occupation and occupational status, income and wealth, and geographical features have often been used as measures of SES [7–9]. SES is often investigated in health disparities research, but as of yet, not much is known about the relationship between socioeconomic factors and low back pain (LBP). Results of the few studies that have reported on this relationship are conflicting [10, 11]. Considering that LBP is one of the most prevalent disorders worldwide, and has an enormous economic and societal burden, it is important to identify factors that might help reduce the prevalence of LBP and its associated disability [12, 13].

Evidence indicates that staying active during a LBP episode leads to quicker recovery and clinical practice guidelines consistently recommend remaining active despite LBP [14]. Disability among adults with LBP has been found to be associated with maladaptive LBP beliefs, such as the belief that movement will cause physical damage to the spine and that bed rest is needed for managing LBP [15–18]. Indeed, beliefs about LBP in the general public tend to be maladaptive [19–24]. This has led developers of mass media interventions to focus on changing beliefs about activity in the hope that it will lead to more adaptive beliefs that change associated health behaviour (i.e. more activity during LBP episodes and thus less disability). Unfortunately, most mass media campaigns have resulted in improvements in beliefs, but have failed to result in sustainable changes in associated disability behaviours [25–27]. Various explanations have been suggested, many of which were campaign-related.

One factor that may have influenced the success of LBP campaigns is SES. Personal and environmental factors play a role in health behaviour, as many health models have suggested. For example, education has been found to be related to LBP beliefs [28]. Individuals with a higher level of education may be more adept at sifting through the vast amount of information about LBP and adopting more adaptive beliefs about their LBP (i.e. believing that one should stay active during an episode of LBP) [29, 30]. Since none of the previous mass media campaigns have examined the relationsship between SES and campaign effects, it is possible that more targeted messaging could result in better outcomes. This study examined the relationship between measures of SES and the belief that one should stay active through LBP among the general population of Alberta, Canada. We also examined the association between measures of SES and exposure to a mass media campaign aimed at improving back beliefs.

## Methods

### Design

Over the past decade, the Workers’ Compensation Board and partners in the province of Alberta, Canada have undertaken a mass media campaign designed to improve back beliefs among the general public. This campaign was evaluated in a previous study [25]. From 2010 through 2014, annual data on public beliefs continued to be gathered using cross-sectional surveys. These surveys contained the key question related to beliefs about staying active during LBP that was also used during the initial study [25]. The Workers’ Compensation Board-Alberta provided access to the survey data that contained measures of LBP beliefs along with several demographic characteristics. Informed consent was not written, but obtained by the polling firms verbally for telephone surveys and online for web-based surveys. Consent was also implied through completion of the survey. The University of Alberta’s Health Research Ethics Board approved this study.

### Study population

Between 2010 and 2014, 9572 randomly selected Alberta residents aged 18–65 years were surveyed. The sample appeared representative of the overall population of adult Albertans based on a comparison with region, sex, and age information available in the most recent Statistics Canada census information [31]. Experienced polling firms collected data using Computer-Assisted Telephone Interviews (*n* = 4500) and web-based surveys (*n* = 5072). The telephone surveys were conducted in January 2010 (*n* = 900), January 2011 (*n* = 900), July 2013 for the year 2012 (*n* = 900), November 2013 for the year 2013 (*n* = 900) and May 2014 (*n* = 900). Web-based surveys were conducted in January 2010 (*n* = 1002), January 2011 (*n* = 1066), July 2013 for the year 2012 (*n* = 1002), November/December 2013 for the year 2013 (*n* = 1001) and May 2014 (*n* = 1001). Respondents to the telephone interviews were randomly selected while the web-based surveys were not random (i.e. self-selected respondents to the online survey). Since the majority of respondents were self-selected, we were unable to calculate response rates.

### Outcome measures

The surveys contained the key belief question from the original campaign surveys regarding staying active with back pain. Respondents were asked their level of agreement (from 1-Completely Disagree to 5-Completely Agree) with the statement “If you have back pain you should try to stay active”. Level of agreement with this statement was considered the primary outcome measure for the current study, and responses were dichotomized by combining the agree options (4 and 5) into one category and disagree options (1 and 2) into a second category with the neutral option (3). Agreement with this statement was considered an adaptive back belief, while not agreeing with the statement was considered a maladaptive back belief. Although there has been little formal reliability or validity testing of this outcome measure of beliefs about LBP, the previous study by Gross et al. highlights the validity of the belief question [25]. It has been used in research performed in Scotland and Canada, and in both locations it was capable of detecting changes in general public back pain beliefs [25, 26]. The surveys also inquired about respondents’ exposure to campaign messaging, asking whether they recalled seeing or hearing any advertising that says “Back pain: Don’t take it lying down” or advising that it is important to stay active through back pain. This self-reported exposure to campaign messaging was considered a secondary outcome measure for this study.

Available measures of SES that were used as independent variables were region of residence (Edmonton/Calgary or other region in Alberta, where Edmonton/Calgary were considered urban and other regions were considered rural), the language respondents first learned at home in their childhood (French/English or other, where French and English were considered native and other languages were considered immigrant), level of education, employment situation, occupation, marital status, income category, and the number of people in the respondents’ household. Education, employment, occupation, and marital status had multiple categories and were collapsed into variables with more meaningful categories for analysis. The specific categories used can be seen in Table 1. Furthermore, the surveys contained basic descriptive information regarding characteristics of the study population. This included type of survey (phone- or web-based), age category, and sex of respondents. The original survey items used in this study are shown in Additional file 1.

**Table 1:** Population Characteristics (*n* = 9572)

Characteristic	N (%)
Age category	
18–24	447 (4.9)
25–34	1175 (12.9)
35–39	933 (10.2)
40–44	1384 (15.2)
45–54	1912 (21.0)
55–64	1891 (20.7)
65+	1375 (15.1)
Not reported	455
Occupation	
Manual workers	864 (9.7)
Office workers	1636 (18.4)
Sales & services workers	816 (9.2)
Professional, Science & Technology workers	1637 (18.4)
Other (Homemaker/Student/Retired/Unemployed)	3928 (44.2)
Not reported	691
Income category	
$ 19.999 or less	445 (5.9)
$ 20.000–39.999	1124 (14.9)
$ 40.000–59.999	1344 (17.8)
$ 60.000–79.999	1187 (15.7)
$ 80.000–99.999	1025 (13.6)
$ 100.000 or more	2431 (32.2)
Not reported	2016
Employment	
Employed (full-time or part-time)	2256 (51.7)
Other (Homemaker/Student/Retired/Unemployed)	2106 (48.3)
Not reported	5210
Number of people in household, mean (SD)	2.75 (1.49)
Range (Min-Max)	1–13
Not reported	5132
Type of survey	
Phone-based	4500 (47.0)
Web-based	5072 (53.0)
Sex	
Female	5271 (55.1)
Region	
Edmonton/Calgary (Urban)	6417 (67.0)
Language	
English/French (Native)	4679 (92.7)
Not reported	4526
Marital status	
Single	1529 (16.2)
Married/common law union	6430 (68.2)
Other (Divorced/Separated/Widowed)	1473 (15.6)
Not reported	140
Agree with ‘Stay Active’	6471 (67.6)
Not reported	1
Exposed to campaign	2409 (42.2)
Not reported^a^	3868
Education	
High School Diploma or Lower	2557 (27.1)
College or Technical Training	3719 (39.4)
University Education	3168 (33.5)
Not reported	128

^a^Large number of missing data on reported campaign exposure, because this question was not asked in all survey years

### Data analysis

Descriptive demographic and SES characteristics of the sample population were summarized. Logistic regression analysis was performed to identify SES measures associated with back beliefs as well as campaign exposure. For the continuous variable (i.e. number of people in respondents’ household), a test for linearity with the outcome measures was performed first. As the assumption of linearity was not violated, this variable was kept as continuous in all models. All SES variables were entered into univariable models first. Variables that were significant in univariable models were then entered into a final multivariable model. However, in cases where eligible variables were highly correlated, as measured by Cramer’s V (>0.90), only the most significant variable was entered to avoid problems with collinearity. Hosmer-Lemeshow tests were performed to examine goodness-of-fit. For all analyses, significance levels were set to a *p*-value of 0.05. All analyses were conducted using IBM SPSS Statistics 23.0 (Armonk, New York).

## Results

### Sample characteristics

Table 1 provides an overview of the characteristics of the sample population. The majority of respondents were older than 44 years of age, female, had completed the web-based survey, lived in an urban region, and their native language was English and/or French. The average household consisted of 2.7 people, and one third of respondents reported an annual household income of $100,000CDN or more. Furthermore, 67.6% of respondents reported agreeing with the statement *“*If you have back pain you should try to stay active*”*, and 42% of respondents reported being exposed to campaign advertising. Most respondents were married, had completed College or Technical training, and were employed full-time. The types of occupations reported by the respondents varied widely, but most respondents (44.2%) were homemakers, students, retirees, or unemployed.

### Logistic regression modeling ‘stay active’ item

Univariable logistic regression analysis showed statistically significant associations between agreement with the ‘Stay Active’ item, age, and several SES measures. People that learned a language other than English and/or French as their first language were less likely to agree with the item (Odds Ratio (OR) 0.78, 95%-Confidence Interval (CI) 0.63–0.98, *p* = 0.03). A higher educational level was positively related to agreement with the item, and there appeared to be a significant and gradient increase (OR 1.23 and 1.25 for college/technical training and university education respectively, both *p* = <0.001). Homemakers, retirees, students, and unemployed people were less likely to agree with the item than people that were employed either full-time or part-time (OR 0.88, 95%-CI 0.78–1.00), and this was statistically significant (*p* = 0.05). Marital status was also significantly related to agreement with the item. People who were married or in common law union were more likely to agree than single people (OR 1.34, 95%-CI 1.20–1.51, *p* = <0.001), as were people that were divorced, separated, or widowed (OR 1.31, 95%-CI 1.13–1.53, *p* = <0.001). Having an income between $40,000 and $59,999, between $80,000 and $99,999, or >$100,000 led to a significant increase in agreement with the statement, compared to having an income of $19,999 or less. Respective ORs for these income categories were 1.24 (95%-CI 1.00–1.55, *p* = 0.05), 1.41 (95%-CI 1.12–1.78, *p* = 0.004), and 1.66 (95%-CI 1.34–2.05, *p* = <0.001).

Variables entered into the multivariable logistic regression analysis for agreement with the ‘stay active’ statement included age, educational level, employment situation, marital status, and income category. While language was significant in the univariable model, there were insufficient cases in the dataset to run this variable in the multivariable model. The only association that remained significant in multivariable regression was that between agreement with the statement and income (*p* = 0.005). A Hosmer-Lemeshow test for the multivariable model showed a *p*-value of 0.16 (X^2^ 11.9 with 8 degrees of freedom), suggesting that the data fit the model. Table 2 shows the crude and adjusted associations between the ‘Stay Active’ item and SES-measures.

**Table 2:** Logistic regression modeling for ‘Stay active’ outcome (*n* = 9443)

	Univariable associations				Multivariable associations		
Variable	OR	95%-CI	*p*-value	Included in multivariable model	OR	95%-CI	*p*-value
Age				Yes			
18–24 (reference)	1		0.12		1		0.29
25–34	1.49	1.49–1.49	**<0.001**		1.08	0.69–1.70	0.74
35–39	1.88	1.88–1.89	**<0.001**		1.02	0.63–1.65	0.94
40–44	2.08	2.08–2.08	**<0.001**		1.24	0.77–2.00	0.38
45–54	1.92	1.92–1.92	**<0.001**		1.23	0.79–1.91	0.37
55–64	2.08	2.07–2.08	**<0.001**		1.27	0.81–1.98	0.30
65+	1.80	1.80–1.81	**<0.001**		1.47	0.93–2.31	0.10
Region				No			
Urban (reference)	1						
Rural	1.00	0.91–1.09	0.92				
Language				No^a^			
English/French (reference)	1						
Other	0.78	0.63–0.98	**0.03**				
N people in household	0.97	0.94–1.02	0.22	No			
Educational level				Yes			
High School Diploma or Lower (reference)	1		**<0.001**		1		0.61
College or Technical Training	1.23	1.12–1.37	**<0.001**		1.08	0.89–1.30	0.44
University Education	1.25	1.12–1.40	**<0.001**		1.11	0.90–1.36	0.35
Employment				Yes			
Employed full-time or part-time (reference)	1				1		
Other (Homemaker/Retired/Unemployed/Student)	0.88	0.78–1.00	**0.05**		0.89	0.75–1.06	0.20
Occupation				No			
Manual workers (reference)	1		0.19				
Office workers	1.14	0.95–1.36	0.16				
Sales & Services workers	1.00	0.82–1.23	1.00				
Professional, Science & Technology workers	1.10	0.92–1.31	0.29				
Other (Homemaker/Student/Retired/Unemployed)	1.00	0.85–1.16	0.95				
Marital status				Yes			
Single (reference)	1		**<0.001**		1		0.42
Married/Common Law Union	1.34	1.20–1.51	**<0.001**		1.47	0.90–1.47	0.28
Other (Divorced/Separated/Widowed)	1.31	1.13–1.53	**<0.001**		1.21	0.90–1.63	0.21
Income category				Yes			
$19.999 or less (reference)	1		**<0.001**		1		**0.005**
$20.000–$39.999	1.16	0.92–1.45	0.21		1.04	0.73–1.48	0.84
$40.000–$59.999	1.24	1.00–1.55	**0.05**		1.13	0.79–1.62	0.51
$60.000–$79.999	1.22	0.98–1.53	0.08		1.18	0.81–1.72	0.38
$80.000–$99.999	1.41	1.12–1.78	**0.004**		1.11	0.75–1.64	0.60
$100.000 or more	1.66	1.34–2.05	**<0.001**		1.62	1.12–2.34	**0.01**

^a^Not sufficient cases to run in multivariable model; Bold indicates statistically significant at *p* > 0.05; Hosmer-Lemeshow *p* = 0.16

### Logistic regression modeling for campaign exposure

Table 3 shows the associations between campaign exposure and SES measures. Univariable regression showed a significant association between campaign exposure and a first language other than English/French, where this group was less likely to remember being exposed to campaign advertising than people that learned English and/or French as their first language (OR 1.41, 95%-CI 1.06–1.87, *p* = 0.02). The association between campaign exposure and overall educational level was not significant (*p* = 0.06), while respondents with university education were significantly less likely to remember being exposed to the campaign than respondents with a high school diploma or a lower educational level (OR 1.17, 95%-CI 1.02–1.34, *p* = 0.03). A significant association was also found between occupation and campaign exposure (*p* = 0.003), where respondents who were homemakers, students, retirees, or unemployed were less likely to remember being exposed to the campaign than manual workers (OR 1.24, 95%-CI 1.01–1.52, *p* = 0.04). Lastly, a significant association was observed between age categories and campaign exposure (*p* = <0.001).

**Table 3:** Logistic regression modeling for campaign exposure outcome (*n* = 5622)

	Univariable associations				Multivariable associations^b^		
Variable	OR	95%-CI	*p*-value	Included in multivariable model	OR	95%-CI	*p*-value
Age				Yes			
18–24 (reference)	1		**<0.001**		1		**<0.001**
25–34	0.61	0.46–0.82	**0.001**		0.62	0.45–0.85	**0.003**
35–39	0.69	0.51–0.94	**0.02**		0.73	0.53–1.01	0.06
40–44	0.84	0.63–1.12	0.24		0.92	0.67–1.26	0.60
45–54	0.86	0.65–1.14	0.29		0.95	0.70–1.28	0.72
55–64	1.00	0.76–1.32	1.00		1.04	0.77–1.39	0.82
65+	0.98	0.74–1.30	0.89		0.96	0.71–1.30	0.80
Region				No			
Urban (reference)	1						
Rural	1.12	0.99–1.24	0.07				
Language				No^a^			
English/French (reference)	1						
Other	1.41	1.06–1.87	**0.02**				
N people in household	0.99	0.94–1.04	0.55	No			
Educational level				Yes			
High School Diploma or Lower (reference)	1		0.06		1		**0.05**
College or Technical Training	1.04	0.91–1.18	0.61		1.08	0.94–1.25	0.28
University Education	1.17	1.02–1.34	**0.03**		1.22	1.04–1.43	**0.02**
Employment				No^c^			
Employed full-time or part-time (reference)	1						
Other (Homemaker/Retired/Unemployed/Student)	1.18	1.01–1.37	**0.04**				
Occupation				Yes			
Manual workers (reference)	1		**0.003**		1		0.26
Office workers	0.93	0.74–1.17	0.53		0.91	0.72–1.15	0.43
Sales & Services workers	1.05	0.80–1.36	0.74		1.03	0.79–1.35	0.83
Professional, Science & Technology workers	1.14	0.91–1.43	0.27		1.07	0.84–1.38	0.57
Other (Homemaker/Student/Retired/Unemployed)	1.24	1.01–1.52	**0.04**		1.10	0.89–1.38	0.38
Marital status				No			
Single (reference)	1		0.44				
Married/Common law union	0.97	0.84–1.13	0.70				
Other (Divorced/Separated/Widowed)	1.07	0.89–1.29	0.49				
Income category				No			
$19.999 or less (reference)	1		0.76				
$20.000–$39.999	1.00	0.74–1.36	0.98				
$40.000–$59.999	1.06	0.79–1.42	0.71				
$60.000–$79.999	1.11	0.82–1.50	0.50				
$80.000–$99.999	0.96	0.71–1.31	0.80				
$100.000 or more	0.98	0.74–1.30	0.88				

^a^Not sufficient cases to run in multivariable model; ^b^Hosmer-Lemeshow *p* = 0.404; ^c^Not included in multivariable model due to high correlation with Occupation (Cramer’s V = 0.92); Bold indicates statistically significant at *p* > 0.05

Age, educational level, and occupation were entered into the multivariable logistic regression for campaign exposure. While language was a significant determinant in the univariable model, there were insufficient cases in the dataset to run this variable in the multivariable model. Employment status was not entered into the multivariable model, because it showed a high correlation with occupation (Cramer’s V = 0.92). The associations between campaign exposure and educational level and age remained significant in the multivariable model (*p* < 0.05), while occupation became non-significant. Respondents with university education were significantly less likely to remember being exposed to the campaign than respondents with a high school diploma or lower education (OR 1.22, 95%-CI 1.04–1.43, *p* = 0.02). A Hosmer-Lemeshow test for the multivariable model showed a *p*-value of 0.40 (X^2^ 8.30 with 8 degrees of freedom), again suggesting a good fit of data.

## Discussion

The current study examined associations between various measures of SES and having adaptive beliefs about back pain (i.e. agreeing with the statement ‘If you have back pain you should try to stay active’). We also examined associations between SES measures and self-reported exposure to a mass media campaign highlighting the importance of staying active through an episode of back pain. Results suggest that annual household income, as s measure of SES, was significantly associated with beliefs about LBP. We also observed a significant association between educational level and self-reported exposure to campaign advertising, where respondents with a high level of education were less likely to remember being exposed to campaign advertising (OR 1.22, *p* = 0.02). This may be due to the nature of the advertising, which targeted industries and workplaces with high risk of back pain (e.g. posters were sent out to construction and manufacturing type workplaces, but they were not sent out to offices or workplaces with a high ratio of university educated workers).

The targeted nature of the campaign is an important factor to keep in mind when interpreting our results. It may be that the LBP campaign did not yield any new information for higher educated respondents, which did not trigger them to remember this campaign. Recent studies have shown that tailored messages and targeted campaigns appear to stimulate greater cognitive activity among recipients than messages that are not tailored [32]. Making information relevant to the target audience has a better chance of effectiveness and is more likely to produce positive changes in health-related behavior [32, 33]. However, adequate exposure to the messages remains important, and supportive activities and policies can contribute to better behavioural or cognitive outcomes [33]. Additionally, targeted information campaigns may assist in reducing health disparities because in this study the targeted campaign was recalled more by low SES groups. This suggests the campaign reached and was understood by its intended audience. If low SES patients’ beliefs become more aligned with those of high SES patients, then recovery from LBP may improve to be similar to recovery rates of high SES groups. However, given that other studies have found that changed beliefs do not necessarily lead to changed actions [25–27], more research to help people translate realigned beliefs into new behaviours is needed.

In our univariable models, several SES measures were associated with adaptive back beliefs regarding staying active through LBP. For example, significant associations were observed between the LBP belief item and language, education, employment, and income. Learning a first language other than English or French, which could be interpreted as being immigrant to Canada, was a significant determinant for not agreeing with the ‘stay active’ item. When looking at language as a determinant for racial or immigrant status, our finding is in line with other research that suggests racial and ethnic disparities exist in health care [34, 35]. Specific to pain-related issues, a previous review showed that such disparities are prominent in pain perception, assessment, and treatment of pain, which underlines our finding that immigrant respondents usually are less likely to have adaptive beliefs about staying active through back pain [36]. Education, employment, and income category were also significantly associated with adaptive LBP beliefs in univariable models, which is in line with other research showing that more education is linked to higher income, and that both are associated with better health [37, 38]. Employment status (employed versus unemployed) might be a mediator between education and income, with more education being linked to higher employment rates, and being employed leading to higher income than being unemployed.

Although little is known about the relationship between SES and beliefs about LBP, our results are consistent with a review of survey data from two U.S. national surveys [39]. This study suggests that prevalence of LBP declines with an increase in education and income. Another review on formal education and LBP suggested that low education is associated with longer duration and higher recurrence of LBP [40]. In general, both higher incomes and higher levels of education have been reported as positively associated with healthy behaviour and thus positive health outcomes [6–9, 41]. However, the potential mechanisms through which SES influences LBP are unclear. One explanation might be that higher educational levels are related to healthy behaviour (e.g. exercise habits), less exposure to occupational risk factors (e.g. heavy manual work), and to higher incomes. Higher incomes can further reduce occupational and environmental risk factors (e.g. better living conditions), increase access to preventive health services, and aid in implementing healthy behaviours. However, other known and unknown factors may influence the relationship between SES and LBP, making the understanding of underlying mechanisms difficult. Furthermore, evidence has suggested that correlations between education, income, and other SES measures are modest, and that SES measures are not interchangeable [7]. Different SES factors can affect health at different times in life, on different levels, and through various causal pathways [7]. SES measures can further interact with other respondent characteristics, such as age. For example, older people have had more time to generate wealth than younger people, despite generally reporting lower incomes than younger people due to being retired, unemployed, or unable to enter the labour market. This interplay between various SES and non-SES measures further complicates interpretation of the relationship between SES and health.

Our results were based on a large, population-based sample that appeared representative of the overall target population based on recent census information available from Statistics Canada [31]. This improves generalizability of these results. However, the current study encountered some limitations. Unfortunately, no information on campaign exposure was available for the years 2010 and 2011, and no control group was available from areas without campaign exposure. We also did not have data on self-reported LBP to relate to measures of SES, and future studies should ideally include data on self-reported LBP. Furthermore, while most of the SES measures had many categories, not all categories were meaningfully interpretable and were collapsed. For example, 11 random categories were available for the ‘occupation’ variable, which was subsequently collapsed into 5 meaningful categories. While it has been recommended that researchers use many categories in order to establish knowledge on which SES factors specifically influence health, the categories chosen must be meaningful and preferably based on evidence [7]. Furthermore, the partially non-randomized sample (web-based survey) might have influenced our results and the cross-sectional nature of the surveys prevented examination of individual-level changes in LBP beliefs due to the campaign.

## Conclusion

Individuals with higher annual household income appear more likely to believe that one should stay active during an episode of LBP. Additionally, targeted information campaigns are recalled more by low SES groups and may thus assist in reducing health disparities. More research is needed to fully understand the association between socioeconomic factors and LBP and to target campaigns accordingly.

## Additional file

Additional file 1: Survey questionnaire items. Shows the original survey questions that were used in this study. (DOCX 19 kb)

## Abbreviations

CI: Confidence interval
LBP: Low back pain
OR: Odds Ratio
SES: Socioeconomic status
