# Supplementary material for: Associations between measures of socio-economic status, beliefs about back pain, and exposure to a mass media campaign to improve back beliefs
Source: BMC Public Health. 2017 May 25;17:504. doi: 10.1186/s12889-017-4387-4 (PMC5445411; doi:10.1186/s12889-017-4387-4)
Supplement: Additional file 1: — Survey questionnaire items. Shows the original survey questions that were used in this study. (DOCX 19 kb) [file 12889_2017_4387_MOESM1_ESM.docx]

**Additional file 1. Survey questionnaire items used in analysis**

| Question/Item | Label | Values |
| --- | --- | --- |
| Type of survey (CATI or Web-based) | Survey type | 0. Web-based  1. Phone |
| Region of residence | Region | 1. Edmonton  2. Calgary  3. Other |
| Please indicate whether you agree or disagree with the following statement on a scale of 1 to 5 where 1 means you completely disagree and 5 means you completely agree. ‘If you have back pain you should try to stay active.’ | Staying active | 0. I don’t know 1. Completely disagree  2.  3.  4.  5. Completely agree |
| Have you seen or heard any advertising in radio or television, newspaper or magazine stories, posters, online, or other reminders that say 'Back pain: don't take it lying down?' and advise you that 'it is important to stay active through back pain'? | Exposure to campaign messaging | 1. Yes  2. No  3. I don’t know |
| What is the language you first learned at home in your childhood and that you still understand? | Language | 1. French  2. English  3. Other  4. English and French  5. French and other  6. English and other |
| Including yourself, how many people are there in your household? | Number of people in household | [Continuous] |
| Which category represents the highest level of education you have completed? | Level of education | 1. Elementary (7 years or less)  2. High school, general or professional (8 to 12 years)  3. College pre-university, technical training, certificate (CEP)  4. University certificates and diplomas  5. University Bachelor (including classical studies)  6. University Masters  7. University Doctorate (PhD) |
| Which category best describes your current employment situation? | Employment situation | 1. Employed full-time  2. Employed part-time  3. Homemaker  4. Retired  5. Unemployed  6. Student  7. Other (not specified) |
| What is your current main occupation? | Occupation | 1. Office worker  2. Personnel specialized in sales  3. Personnel specialized in services  4. Manual workers  5. Skilled, semi-skilled workers  6. Science and technology workers  7. Professionals  8. Managers/administrators/owners  9. Homemaker  10. Student (full-time or whose studies take up most of his/her time)  11. Retired (pre-retired or private means)  12. Unemployed (unemployed, welfare)  13. Other |
| Marital status | Marital status | 1. Single  2. Married/common law union  3. Divorced  4. Separated  5. Widowed |
| Which of the following categories reflects the total INCOME before taxes of all members of your household? | Income category | 1. $19,999 or less  2. Between $20,000 and $39,999  3. Between $40,000 and $59,999  4. Between $60,000 and $79,999  5. Between $80,000 and $99,999  6. $100,000 or more |
